# Molecular Characteristics of *Staphylococcus aureus* From Food Samples and Food Poisoning Outbreaks in Shijiazhuang, China

**DOI:** 10.3389/fmicb.2021.652276

**Published:** 2021-06-22

**Authors:** Guoping Lv, Ruiping Jiang, Han Zhang, Lei Wang, Lijie Li, Weili Gao, Hong Zhang, Yantao Pei, Xiuping Wei, Hongyan Dong, Liyun Qin

**Affiliations:** ^1^Basic Medicine College, Hebei University of Chinese Medicine, Shijiazhuang, China; ^2^Shijiazhuang Center for Disease Control and Prevention, Shijiazhuang, China; ^3^College of Life Science, Hebei Normal University, Shijiazhuang, China

**Keywords:** *Staphylococcus aureus*, food poisoning, MLST, antibiotics resistance, staphylococcal enterotoxin

## Abstract

As an opportunistic pathogen worldwide, *Staphylococcus aureus* can cause food poisoning and human infections. This study investigated the sequence typing, the penicillin (blaZ) and methicillin (mec) resistance profiles of *S. aureus* from food samples and food poisoning outbreaks in Shijiazhuang City, and the staphylococcal enterotoxin (SE) types of the *S. aureus* isolates from food poisoning. A total of 138 foodborne *S. aureus* isolates were distributed into 8 clonal complexes (CCs) and 12 singletons. CC1, CC5, CC8, CC15, CC97, CC59, CC398, CC88, and CC7 were the predominant CCs of foodborne *S. aureus* isolates. Moreover, CC59, CC15, and CC5 were the most prevalent CCs in food poisoning outbreaks. SEE was the most commonly detected SE in food poisoning isolates. One hundred thirty-three *S. aureus* isolates harbored the penicillin-resistant gene *blaZ*, and nine isolates carried the *mec* gene. The present study further explained the relationship between *S. aureus* and foods and food poisoning and indicated the potential risk of *S. aureus* infection.

## Introduction

As an important Gram-positive spherical pathogen, *Staphylococcus aureus* can release a variety of heat-stable staphylococcal enterotoxins (SEs) into food (Hennekinne et al., 2012; Wang et al., 2017; Oliveira et al., 2018) responsible for staphylococcal food poisoning (SFP) in human population. SFP is characterized by foodborne gastroenteritis (Argudín et al., 2010; Hennekinne et al., 2012; Lv et al., 2014), and it was recognized as one of the most prominent culprits in food poisoning outbreaks worldwide (Hennekinne et al., 2012; Wu et al., 2016). *S. aureus* can carry multiple SE genes and produce different SEs and SE-like toxins. The SEs (SEA to SEE; SEG to SEI; SEK; SEM to SET) and SE-like toxins (SElJ; SElL; SElU to SElZ) were reported as responsible agents for food poisoning outbreaks (Ono et al., 2008; Argudín et al., 2010; Hennekinne et al., 2012; Lv et al., 2014). SEA, SEB, SEC, SED, and SEE were the most common toxins implicated in SFP (Hennekinne et al., 2012; Kadariya et al., 2014).

Multilocus sequence typing (MLST) analysis of *S. aureus* is important for the prognosis of infection, and it provides means to trace epidemiologically related strains contributing to the tracking of the contamination source. The MLST types of *S. aureus* vary with regions and sources (Pereira et al., 2009; Cheng et al., 2013; Rao et al., 2015; Thapaliya et al., 2017; Htun et al., 2018; Kitti et al., 2018; Wu et al., 2018; Li et al., 2019).

The MLST genotype of *S. aureus* has been reported to influence the complications, severity, and mortality of infection; the strains of CC5 and CC30 exhibited a significant trend toward increased levels of hematogenous complications (Chen et al., 2010). The sequence type 121 of *S. aureus* (ST121) infected the patients who often required long hospitalization and prolonged antimicrobial therapy (Rao et al., 2015), CC398 were associated with high mortality (Bouiller et al., 2016).

*Staphylococcus aureus* can acquire the antibiotic resistance determinants that may confer resistance to many antibiotics. High resistance of *S. aureus* to penicillin was observed (Wu et al., 2018; Li et al., 2019; Dastmalchi Saei and Panahi, 2020); moreover, the resistance to most beta-lactams and the other antibiotics except ceftaroline and ceftobiprole was reported for methicillin-resistant *S. aureus* (MRSA) strains (Lakhundi and Zhang, 2018; Wu et al., 2018; Li et al., 2019). MRSA infections are associated with higher mortality rates than the infections caused by methicillin-susceptible strains. MRSA isolates carry a mobile genetic element of staphylococcal cassette chromosome *mec* (SCC*mec*), the determinant that encodes low-affinity penicillin-binding protein (PBP) similar to PBP2a or PBP2′. The *mec* genes share ≥70% nucleotide sequence identity with the *mecA* gene (Ito et al., 2012; Lakhundi and Zhang, 2018).

This study aimed to characterize *S. aureus* isolates from SFP outbreaks and the food samples isolated in Shijiazhuang, China. In particular, MLST genotypes and the presence of beta-lactam-resistant genes of *blaZ* and *mec* were determined on all isolates. In addition, the enterotoxigenic status of SFP isolates was explored.

## Materials and Methods

### Bacterial Isolates

One hundred and thirty-eight *S. aureus* isolates were used in this study. Eighty-two of the strains were isolated from food samples in 2011–2019, and 56 strains were isolated from 20 SFP outbreaks in 2009–2016. All the isolates were identified as *S. aureus* using conventional microbiological methods including Gram staining and catalase and coagulase tests and then stored in a Brain Heart Infusion (BHI) medium with 40% glycerine at −80°C. Species was confirmed by Matrix-Assisted Laser Desorption Ionization Time of Flight Mass Spectrometry (MALDI-TOF MS) System (Bruker, Berlin, Germany) before further experiments.

### Primers

All the primers used in the study were synthesized by TaKaRa (TaKaRa, Beijing, China) (Table 1). Three pairs of primers of *mec*, *blaZ*, and *nu*cA were designed using the PrimerSelect program of the DNASTAR software according to the reported sequences from the Gene Databank (NCBI, NIH, Bethesda, MD, United States). The primers of MLST were available from the MLST website^1^.

**TABLE 1:** Primers used in the study.

Primers	Nucleotides Sequence(5′–3′)	Target gene	Amplicon size (bp)
*mec*-F	ACCACCCAATTTGTCTGCCAGTT	*mec*	800
*mec*-R	TGGCTCAGGTACTGCTATCCACCCC		
*blaZ*-F	CAAAGATGATATAGTTGCTTATTCTCC	*blaZ*	421
*blaZ*-R	TGCTTGACCACTTTTATCAGC		
*nucA*-F	CGCTTGCTATGATTGTGGTAGCC	*nucA*	126
*nucA*-R	TTCGGTTTCACCGTTTCTTGGCG		
*arcC*-F	TTGATTCACCAGCGCGTATTGTC	*arcC*	456
*arcC*-R	AGG TATCTGCTTCAATCAGCG		
*aroE*-F	ATCGGAAATCCTATTTCACATTC	*aroE*	456
*aroE*-R	GGTGTTGTATTAATAACGATATC		
*glpF*-F	CTAGGAACTGCAATCTTAATCC	*glpF*	465
*glpF*-R	TGGTAAAATCGCATGTCCAATTC		
*gmk*-F	ATCGTTTTATCGGGACCATC	*gmk*	417
*gmk*-R	TCATTAACTACAACGTAATCGTA		
*pta*-F	GTTAAAATCGTATTACCTGAAGG	*pta*	564
*pta*-R	GACCCTTTTGTTGAAAAGCTTAA		
*tpi*-F	TCGTTCATTCTGAACGTCGTGAA	*tpi*	402
*tpi*-R	TTTGCACCTTCTAACAATTGTAC		
*yqiL*-F	CAGCATACAGGACACCTATTGGC	*yqiL*	516
*yqiL*-R	CGTTGAGGAATCGATACTGGAAC		

### Staphylococcus Enterotoxin Detection

Staphylococcal enterotoxins of the SPF isolates were detected by ELISA using a RIDASCREEN SET A, B, C, D, E assay kit (R-Biopharm, Pfungstadt, Germany). *S. aureus* isolates were pre-enriched in BHI broth, and the supernatant of 24-h cultures of one colony grown in BHI at 37°C was centrifuged at 6,000 × *g* for 10 min at 4°C. The supernatant was aseptically filtered by a 2.0-μm Millipore filter, and the filtered sample was added into an ELISA plate. The enterotoxins were tested according to the protocol recommended by the manufacturer’s instructions. The absorbance was measured at 450 nm in the Epoch ELISA reader (BioTek, Winooski, VT, United States).

### DNA Extraction

*Staphylococcus aureus* isolates were grown overnight in BHI broth at 37°C. Genomic DNA was extracted using a bacterial genomic DNA extraction kit (DNeasy Blood and Tissue Kit, Qiagen Inc., Redwood City, CA, United States) according to the manufacturer’s instructions.

### Detection of *mec* and *blaZ*

All the isolates underwent antimicrobial resistance gene typing. The presence of two antimicrobial resistance genes of *blaZ* and *mec* was assessed together with the reference gene of *nucA*, using a multiplex PCR assay. The three pairs of primers used are listed in Table 1. The PCR mixture contained 4 μl *mec*-F (2.5 μM), 4 μl *mec*-R (2.5 μM), 4 μl *blaZ*-F (2.5 μM), 4 μl *blaZ*-R (2.5 μM), 4 μl *nucA*-F (2.5 μM), 4 μl *nucA*-R (2.5 μM), 2.5 μl genome DNA template, 5 μl 10 × Ex Taq buffer (Mg2^+^ plus) (20 mM), 4 μl dNTP mixture (2.5 mM each), 0.5 μl TaKaRa Ex Taq DNA polymerase (TaKaRA, China), and 14 μl double-distilled water. The PCR conditions were as follows: initial denaturation at 94°C for 5 min, followed by 35 cycles of denaturation at 94°C for 30 s, annealing at 68°C for 60 s, extension at 72°C for 60 s, and a final extension at 72°C for 10 min. All the PCR products were analyzed by QIAxcel system (Qiagen, Germany). Separation was performed using the AM320 method (100 ng/μl sample was injected at the voltage of 5 kV for 10 s and separated at the voltage of 6 kV for 320 s). The samples with two fragments were considered positive to *nucA* and *mec* or *blaZ* according to the fragment size, and the samples with three fragments were positive to *nucA*, *mec*, and *blaZ*. The *mec* and *blaZ* PCR fragments were sequenced using Sanger dideoxy DNA sequencing (Sangon, Shanghai, China), and the sequences were blasted in NCBI BLAST web.

### MLST of *S. aureus* Isolates

Seven housekeeping genes of *S. aureus of arcC*, *aroE*, *glpF*, *gmk*, *pta*, *tpi*, and *yqiL* were adapted for MLST. These seven housekeeping genes were amplified by PCR according to the original and alternative protocols available from the MLST website (see text footnote 2). The amplicons were sequenced using Sanger dideoxy DNA sequencing (Sangon, China). The sequences were compared with the known alleles in the MLST database to determine the allele number, ST types, and CCs. The isolates that did not match with the previously identified STs were submitted to the database and assigned as new STs. The phylogenetic tree was constructed using BioNumerics 7.6.2 (Applied Maths, Sint-Martens-Latem, Belgium).

## Results

### Presence of *mec* and *blaZ*

Of the 138 *S. aureus* isolates, 133 isolates harbored the penicillin-resistant gene of *bla*Z *and* nine isolates harbored the methicillin-resistant gene of *mec* (Table 2). Four *blaZ*-negative isolates were isolated from food samples, and one *blaZ* negative isolate was isolated from SFP outbreaks. The nine *mec*-positive isolates originated from four SFP outbreaks, and five *mec*-positive isolates originated from food samples (four cooked foods and one hamburger).

**TABLE 2:** Resistance gene characterization of *S. aureus* isolates.

Genes	Isolates of food samples				Foodborne outbreak isolates			
	*mec*+	*mec*-	*bla*Z+	*bla*Z-	*mec*+	*mec*-	*bla*Z+	*bla*Z-
Number	5	77	78	4	4	52	55	1
%	6.1	93.9	95.1	4.9	7.1	92.9	98.2	1.8

### STs and CCs of *S. aureus* Isolates

**FIGURE 1:**
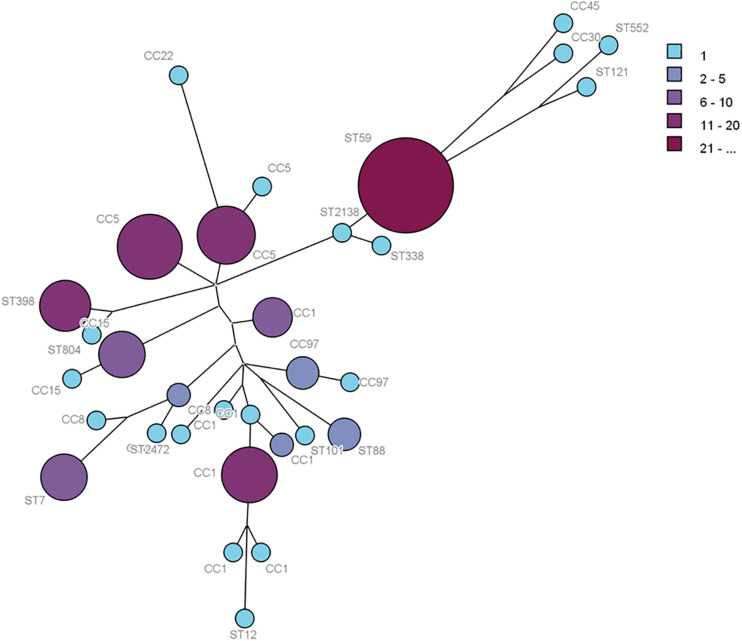
Phylogenetic tree of 138*S. aureus* isolates based on MLST allelic profiles. Each circle represents CC type or singleton, and the size and color depth of each circle correspond to the number of samples (1, 2–5, 6–10, 11–20, and ≥21).

Multilocus sequence typing analysis allowed the distribution of the isolates in CCs, defined as those STs that matched the central genotype, and singletons that differed from all STs in the dataset and did not belong to any CC. The 138 foodborne *S. aureus* isolates, 82 isolates from food samples, and 56 isolates from food poisoning outbreak were respectively distributed into 31 distinct STs, 25 STs, and 15 STs. The 31 STs were divided into eight CCs by eBURST, and 12 STs were singletons. The minimum spanning network was built from the core genome MLST allelic profiles of 138 *S. aureus* isolates with CCs and singletons (Figure 1). The CCs of cladogram showed high diversity. The most prevalent foodborne *S. aureus* isolates were CC1, CC5, CC8, CC15, CC97, CC59, CC398, CC88, and CC7; other clonal complexes were isolated sporadically (Figure 1).

In SFP isolates, ST59 was the most prevalent type (32.1%, *n* = 18), followed by ST6 (25.0%, *n* = 14), ST15 (10.7%, *n* = 6), ST5 (8.9%, *n* = 5), and ST398 (5.3%, *n* = 3). The other 10 STs, including ST9, ST2791, ST72, ST1003, ST30, ST464, ST7, ST88, ST338, and ST804, accounted for 1.8% (*n* = 1), respectively (Table 3).

**TABLE 3:** Molecular characteristics of STs in foodborne *S. aureus*.

CCs	STs number	Food								Foodborne outbreak
		Cooked food	Juices	Milk	Bread	Hamburger	Snacks	Raw meats	Frozen food	
CC1	ST1	4	1	0	0	0	0	3	4	0
	ST9	2	0	0	0	0	0	2	2	1
	ST188	2	0	0	0	0	0	0	0	0
	ST1920	1	0	0	0	0	0	0	0	0
	ST2139	0	0	0	1	0	0	0	0	0
	New	0	0	0	0	1	0	0	0	0
	ST2791	0	0	0	0	0	0	0	0	1
CC5	ST5	7	0	0	0	2	0	0	0	5
	ST6	3	0	0	0	0	0	1	0	14
	ST965	0	0	0	0	0	1	0	0	0
CC8	ST72	0	0	0	0	0	0	0	0	1
	ST630	0	0	0	0	0	2	0	0	0
	ST1821	1	0	0	0	0	0	0	0	0
CC15	ST15	3	0	0	0	0	0	0	0	6
	ST1003	0	0	0	0	0	0	0	0	1
CC22	ST22	0	0	0	0	0	0	0	1	0
CC30	ST30	0	0	0	0	0	0	0	0	1
CC45	New	1	0	0	0	0	0	0	0	0
CC97	ST464	4	0	0	0	0	0	0	0	1
ST7	ST7	3	0	1	1	0	0	1	2	1
ST12	ST12	0	0	0	0	0	0	0	1	0
ST59	ST59	3	2	0	0	0	1	2	0	18
ST88	ST88	1	0	0	0	0	0	0	2	1
ST101	ST101	0	0	0	0	0	0	1	0	0
ST121	New	0	0	1	0	0	0	0	0	0
ST338	ST338	0	0	0	0	0	0	0	0	1
ST398	ST398	4	0	2	0	0	1	1	0	3
ST552	ST552	0	0	0	0	0	0	1	0	0
ST804	ST804	0	0	0	0	0	0	0	0	1
ST2138	ST2138	1	0	0	0	0	0	0	0	0
ST2562	ST2562	0	0	0	0	0	0	1	0	0
Total		40	3	4	2	3	5	13	12	56

**TABLE 4:** SE characteristics and the related STs of SFP *S. aureus* isolates.

SE types	Number	STs
SEA-SED-SEE	14	ST6
SEA-SEB-SEC	13	ST59
SEE	6	ST15
	1	ST464
	1	ST7
	1	ST72
	1	ST88
SEB-SEC	5	ST59
	1	ST338
SEC-SED-SEE	5	ST5
SEA-SEE	1	ST1003
SEA-SEB-SED-SEE	1	ST30
–	3	ST398
	1	ST9
	1	ST2791
	1	ST804

Eighty-two *S. aureus* isolates were identified from eight kinds of food samples (Table 3). ST1 was the most prevalent isolate (14.6%, *n* = 12), followed by ST5 (11.0%, *n* = 9), ST59 (9.8%, *n* = 8), ST398 (9.8%, *n* = 8), ST7 (9.8%, *n* = 8), ST9 (7.3%, *n* = 6), ST6 (4.9%, *n* = 4), ST464 (4.9%, *n* = 4), ST15 (3.7%, *n* = 3), ST88 (3.7%, *n* = 3), and ST188 (2.4%, *n* = 2). Other STs were isolated sporadically (Table 3), including ST12, ST22, ST101, ST552, ST2138, ST2139, ST2462, ST1821, ST965, and ST1930 and three new STs. Twenty-five STs were divided into 7 CCs and 10 singletons (Table 3). Twelve ST1 isolates originated from four kinds of food samples (four from cooked food, four from frozen food, three from meat, and one from juice). Nine ST5 isolates were taken from two kinds of food samples (seven from cooked food and two from hamburgers). The distribution of the other STs is shown in Table 3.

### Production of Enterotoxins in Isolates of SFP Outbreaks

The production of SEA, SEB, SEC, SED, and SEE was evaluated for the 56 *S. aureus* isolates from food poisoning outbreaks, and eight distinct SE profiles were identified (Table 4). The most prevalent SEA-SED-SEE accounted for 25.0% (14/56), followed by SEA-SEB-SEC 23.2% (13/56), SEE 17.9% (10/56), SEB-SEC 10.7% (6/56), SEC-SED-SEE 8.9% (5/56), SEA-SEE 1.8% (1/56), and SEA-SEB-SED-SEE 1.8% (1/56). SEA, SEB, SEC, SED, and SEE were not detected in six strains.

## Discussion

Staphylococcal food poisoning outbreaks are often caused by foodborne *S. aureus*. The CCs of SFP isolates have their distribution characteristics. In this study, the isolates were assigned to 12 CCs in SFP outbreaks, including CC5, CC59, CC15, CC30, CC8, CC1, CC97, CC38, CC398, CC7, CC804, and CC88. Among them, CC59, CC15, CC5, and CC398 were the predominant CCs. While in Tokyo, Japan, CC8, CC6, CC5, CC508, CC59, CC20, and CC30 were the prevalent CCs of the SFP isolates in the descending order (Suzuki et al., 2014). Some epidemic CCs of SFP isolates were also the prevalent CCs in foods and hospitals. CC1, CC5, CC8, CC15, CC97, CC7, CC59, and CC398 were the most prevalent clonal complexes in food samples in this study. CC5, CC8, CC188, CC59, CC7, and CC398 were the most prevalent CCs in the teaching hospitals of China (Li et al., 2018). The high prevalence of CC59, CC15, CC5, and CC398 was related with the SFP outbreaks. These CCs are common in China and often detected in foods, animals, and humans (Bi et al., 2018; Li et al., 2018; Peng et al., 2018; Wu et al., 2018). The *S. aureus* strains from humans and animals can contaminate foods; they are also a major cause of food contamination.

The SE produced by *S. aureus* is the direct cause of SFP. In this study, eight distinct SE types were identified from food poisoning isolates. SEE was the most commonly detected SE in SFP isolates, followed by SEA, SEC, SEB, and SED. SEA, SEB, SEC, SED, or SEE was not detected in several isolates, but other types of SEs could be produced by these strains, so they should be analyzed by more sensitive methods such as SE gene detection. Meanwhile, the assay kit should be improved to detect all types of SEs and SE-like toxins in the future, because many other SEs and SE-like toxins can also cause food poisoning outbreaks. In this study, there were some correlations between STs and SE types in the SFP isolates. The isolates of ST15, ST6, and ST5 were respectively of SEE, SEA-SED-SEE, and SEC-SED-SEE types. However, the ST59 isolates in food poisoning outbreaks were of SEA-SEB-SEC or SEB-SEC types (Table 4). The SE characteristics of the strain might be related with its region and source. So, more literature data is needed to prove the correlations.

The prevalence of foodborne *S. aureus* would increase the risk of causing human infection and food poisoning. The STs of epidemic *S. aureus* in foods were respectively ST1, ST5, ST59, ST398, ST7, ST9, ST6, ST464, ST15, and ST88 in descending order. ST1, ST5, ST6, ST7, ST9, ST15, ST59, and ST398 were the common STs of foodborne isolates in China (Wu et al., 2018; Li et al., 2021). They had different characteristics in SFP outbreaks in this study. As the low-risk SFP isolates, ST1, ST7, and ST9 are widely distributed in foods, but ST1 isolates were not involved in any SFP outbreaks, and ST7 and ST9 isolates rarely caused food poisoning. ST6 and ST15 were the highly frequent isolates in SFP outbreaks, whereas ST5, ST59, and ST398 isolates were the highly frequent isolates in SFP outbreaks and food contamination.

ST464 was the main isolate in cooked food, and it caused one SFP outbreak. The other dominant clones were ST5, ST398, ST1, ST15, and ST59. ST1, ST9, and ST59 were the dominant clones from raw meat in this study. With the exception of ST464, these STs were the prevalent isolates in retail meat and meat products of China (Wu et al., 2018). ST398, ST15, and ST59 were also the main STs from sushi samples in Beijing, China (Li et al., 2021). The ST5, ST15, and ST398 isolates were also the prevalent foodborne *S. aureus* in other countries (Thapaliya et al., 2017; Li et al., 2019). ST1, ST5, ST15, ST59, and ST398 were also included in hospitals, community, and animals (Li et al., 2017, 2018; Bi et al., 2018; Peng et al., 2018; Heaton et al., 2020). The pollution of these isolates was closely related to animal and human activities. The transmissions of these strains also increased the chance of acquiring drug resistance.

Nine MRSA isolates were obtained from foodborne *S. aureus* isolates. ST15, ST59, and ST338 were the ST types of four MRSA isolates from SFP outbreaks, and ST5, ST15, and ST59 were the ST types of the other five MRSA isolates from food samples. Two MRSA ST59 isolates were detected in sushi samples from Beijing, China (Li et al., 2021). ST5, ST15, and ST59 were also the ST types often detected among iatrogenic isolates. They were the prevalent isolates from healthcare-associated MRSA (HA-MRSA) and community-associated MRSA (CA-MRSA) of China (Li et al., 2018; Peng et al., 2018; Wu et al., 2018; Yuan et al., 2019). The contamination of these STs in food was associated with human activities. The proportion of MRSA isolates in foodborne *S. aureus* strains was 6.5% in this study. The low contamination rate of foodborne MRSA has been reported in China. MRSA was present in 7.9% of 1,150 *S. aureus* isolates from retail food and approximately 7.1% of *S. aureus* isolates from meat and meat products (Wang et al., 2017; Wu et al., 2018). The proportion of MRSA in hospitals and healthy human carriers was higher than that of the foodborne *S. aureus* in China. The MRSA accounted for 36.5% in teaching hospitals (Li et al., 2018), 44.7% in clinical *S. aureus* (Yuan et al., 2019), and 14.8% among the healthy adult carriers (Fan et al., 2016). The prevalence of foodborne MRSA is due to the human activity that spreads the bacteria through the food chain. It is possible that the transmissions between humans and foods are bidirectional.

In this study, 96.4% (133/138) of the foodborne *S. aureus* isolates contained the *blaZ* gene-encoding penicillin resistance. The resistance of *S. aureus* to penicillin was prevalent in China, which respectively accounted for 84.6% (735/868) in meat samples (Wu et al., 2018), 83.7% (963/1150) from retail foods (Wang et al., 2017), and 90.7% (98/108) among healthy adult carriers (Fan et al., 2016). The penicillin resistance rate of *S. aureus* in China was higher than that in other countries. Seventy-three percent (108/148) of the *S. aureus* strains from food products were resistant to penicillin in Portugal (Pereira et al., 2009) and 56.8% (50/88) in Denmark (Li et al., 2019). The prevalence of penicillin-resistant strains of foodborne *S. aureus* increases the risk of infection in humans.

*Staphylococcus aureus* can spread through the food chain. As the most prevalent foodborne ST, ST1 was also included in humans and animals and could cause infections at some time (Chen et al., 2017; Li et al., 2017; Peng et al., 2018; Wu et al., 2018). As the common foodborne STs, ST6 and ST7 were included in the transmission in communities and hospitals (Fan et al., 2016; Li et al., 2018; Peng et al., 2018). ST59 and ST5 were the main pathogenetic isolates and the predominant clonal lineages in community and healthcare in China (Bi et al., 2018; Li et al., 2018; Peng et al., 2018; Yuan et al., 2019). ST59 was the common food-related MRSA in China (Wu et al., 2018). ST398 and ST9 were the main livestock-associated *S. aureus* and the prevalent isolates to cause bovine mastitis in China (Yan et al., 2014; Li et al., 2017; Bi et al., 2018). ST398 was also common in communities and hospitals (Li et al., 2018; Peng et al., 2018). These epidemic isolates are widely distributed in the environment including humans, animals, and foods. Their transmissions in the environment have epidemiological correlation. It is important for public health to control the transmissions of foodborne *S. aureus*.

## Conclusion

This study provides the epidemic characteristics of foodborne *S. aureus* isolates in Shijiazhuang, China. ST1 was the predominant isolate in food samples, followed by ST5, ST7, ST59, ST398, ST9, ST6, and ST464. Moreover, ST59 was the dominant ST in food poisoning outbreaks, followed by ST6, ST15, ST5, and ST398. The food poisoning isolates can produce one or more SEs. SEE was the most commonly detected SE in food poisoning isolates. Most of the foodborne *S. aureus* isolates contained the *blaZ* gene, and several isolates were MRSA. The prevalence and transmission of foodborne *S. aureus* will increase the risk of infection. These data could help us understand the contamination and transmission of this bacterium.

## Data Availability Statement

The original contributions presented in the study are included in the article/supplementary material, further inquiries can be directed to the corresponding authors.

## Author Contributions

GL and LQ performed the research. GL and RJ analyzed the data and wrote the manuscript. HaZ performed the blaZ and mec testing and analysis. RJ, LW, HoZ, and YP contributed to the MLST analysis. LL, WG, XW, and HD performed the detection of staphylococcus enterotoxins. All authors have approved the manuscript and agreed with submission to your esteemed journal.

## Conflict of Interest

The authors declare that the research was conducted in the absence of any commercial or financial relationships that could be construed as a potential conflict of interest.

## Footnotes

1 http://saureus.mlst.net
